# Association between *Salmonella* infection and colon cancer: a nationwide registry-based cohort study

**DOI:** 10.1017/S0950268821000285

**Published:** 2021-02-08

**Authors:** Janneke W. Duijster, Jørgen V. Hansen, Eelco Franz, Jacques J. C. Neefjes, Morten Frisch, Lapo Mughini-Gras, Steen Ethelberg

**Affiliations:** 1 Centre for Infectious Disease Control, National Institute for Public Health and the Environment (RIVM), Bilthoven, the Netherlands; 2 Department of Cell and Chemical Biology, Oncode Institute, Leiden University Medical Center (LUMC), Leiden, the Netherlands; 3 Department of Epidemiology Research, Statens Serum Institut, Copenhagen, Denmark; 4 Institute for Risk Assessment Sciences, Utrecht University Medical Center, Utrecht, the Netherlands; 5 Department of Infectious Disease Epidemiology and Prevention, Statens Serum Institut, Copenhagen, Denmark; 6 Global Health Section, Department of Public Health, University of Copenhagen, Kobenhavn, Denmark

**Keywords:** Colon cancer, inflammatory bowel disease, non-typhoidal *Salmonella*, salmonellosis

## Abstract

Laboratory data increasingly suggest that *Salmonella* infection may contribute to colon cancer (CC) development. Here, we examined epidemiologically the potential risk of CC associated with salmonellosis in the human population. We conducted a population-based cohort study using four health registries in Denmark. Person-level demographic data of all residents were linked to laboratory-confirmed non-typhoidal salmonellosis and to CC diagnoses in 1994–2016. Hazard ratios (HRs) for CC (overall/proximal/distal) associated with reported salmonellosis were estimated using Cox proportional hazard models. Potential effects of serovar, age, sex, inflammatory bowel disease and follow-up time post-infection were also assessed. We found no increased risk of CC ≥1 year post-infection (HR 0.99; 95% confidence interval (CI) 0.88–1.13). When stratifying by serovar, there was a significantly increased risk of proximal CC ≥1 year post-infection with serovars other than Enteritidis and Typhimurium (HR 1.40; 95% CI 1.03–1.90). CC risk was significantly increased in the first year post-infection (HR 2.08; 95% CI 1.48–2.93). The association between salmonellosis and CC in the first year post-infection can be explained by increased stool testing around the time of CC diagnosis. The association between proximal CC and non-Enteritidis/non-Typhimurium serovars is unclear and warrants further investigation. Overall, this study provides epidemiological evidence that notified *Salmonella* infections do not contribute significantly to CC risk in the studied population.

## Introduction

Colon cancer (CC) is the third most common cancer in industrialised countries, with 1.1 million new diagnoses annually worldwide [1]. Although genetic, environmental and lifestyle-related exposures are the best-known risk factors for cancer, around 20% of the global cancer burden is estimated to be attributable to infectious agents, including bacteria [2]. Examples hereof concerning the gastrointestinal tract include *Helicobacter pylori* infection as risk factor for gastric cancer, and *Salmonella* Typhi infection as risk factor for gallbladder carcinoma in chronic typhoid carriers [3–5].

Several mechanisms have been identified through which bacteria can contribute to cancer formation. These include chronic inflammation, production of DNA-damaging toxins and manipulation of host cell signalling pathways [3, 4, 6]. The latter promotes bacterial uptake, intracellular survival and egress in case of *Salmonella* infection. Indeed, several *Salmonella* effector proteins have been shown to activate the major host cell signalling pathways AKT and MAPK, which are central to many signalling cascades and are often deregulated in cancers [4]. *Salmonella* is expected to contribute to carcinogenesis mainly under conditions of long-lasting infections, an intact bacterial type 3 secretion System (T3SS), and with a background of host predisposition, in which significant numbers of pre-transformed cells are present in the intestine. This has been shown *in vivo* by experiments demonstrating a higher risk of colon carcinoma formation after infection with wild type *vs.* Δ*prgH* mutant *S*. Typhimurium (lacking the T3SS) strains in mice genetically predisposed to cancer (APC+/−) *vs.* normal mice [4].

Against this background of experimental data, population-based epidemiological studies addressing the association between *Salmonella* infection and CC are limited to one [7]. In a nationwide registry study in the Netherlands, an increased risk of CC was observed among patients who had a reported (severe) *Salmonella* infection between 20 and 60 years of age as compared to the baseline CC risk in the Dutch population [7]. This increased risk was significant following infection with *S*. Enteritidis and for the proximal part of the colon. Moreover, it was shown that among CC patients, the risk of having had a previously notified *Salmonella* infection was higher for individuals with pre-infectious inflammatory bowel disease (IBD), although numbers were small [7]. IBD is a known risk factor for both CC and salmonellosis, as this chronic condition is associated with recurrent episodes of gut inflammation and increased susceptibility to infection and testing [8, 9].

*Salmonella* is a major cause of bacterial gastroenteritis worldwide, with over 90 000 infections reported to public health authorities in Europe each year [10]. In Denmark, an annual average of 1100 salmonellosis cases has been reported in recent years through the national surveillance system [11]. As most *Salmonella* infections are mild with self-limiting symptoms, the majority of infections go unreported. It is estimated that the true number of *Salmonella* infections (i.e. after correction for underreporting and underdiagnosis) is approximately 10 times higher than the number of infections reported in the national disease surveillance system in Denmark [12]. Each year, around 3400 people are diagnosed with CC in the Danish population [13]. Although screening programmes aiming at early detection of CC typically target the older population (i.e. individuals aged >50 years), the incidence of CC in young adults has increased during the last 25 years, being a cause for concern [14].

In this study, we assessed the potential association between *Salmonella* infection and CC in Denmark. To this end, we made use of data from comprehensive health registries in Denmark to compare the incidence of CC among individuals with a previously reported salmonellosis to that of individuals without reported salmonellosis. In addition, we assessed potential effects in subgroup analyses as defined by age, sex, IBD and time since infection on the association between *Salmonella* infection and CC.

## Methods

### Data sources

We conducted a population-based cohort study with data from four health registries in Denmark between January 1994 and December 2016. Demographic characteristics including sex, date of birth, vital status (e.g. date of death, immigration and emigration), marital status and region of living of all people residing in Denmark were retrieved from the Danish Civil Registration System [15]. A second dataset included information on bacterial gastrointestinal infections, with recorded bacterial species and subspecies/serovar and date of diagnosis (Danish Register on Enteric Pathogens) [16]. The presence of IBD, i.e. ulcerative colitis and Crohn's disease with date of diagnosis, was obtained from the Danish National Patient Registry [17]. The fourth dataset contained all CC diagnoses from 1978 until December 2016 reported to the Danish Cancer Registry, with date of diagnosis and tumour location (based on ICD-10 code) [18]. Data of all four sources were matched using the CPR-number, which is a unique identifier used across all national registries [15].

### Study population

The cohort consisted of 7 646 978 individuals who contributed at least 1 day of follow-up between 1994 and 2016, of which 47 856 had been diagnosed with a *Salmonella* infection. Median age at infection was 34 years (interquartile range (IQR): 14–54). *S*. Enteritidis (*S*E) (43.5%) and (monophasic) *S*. Typhimurium (*S*T) (28.6%) caused the majority of reported infections. Among the more than 400 other reported serovars (hereafter referred to as ‘other serovars’), *S*. Infantis, *S*. Newport and *S*. Stanley were the most frequent.

### Exposure and outcome definition

The exposure variable was defined as having or not having had a reported non-typhoidal *Salmonella* infection. *Salmonella* infection was categorised into infections with *S*E, *S*T or other serovars. For individuals with multiple *Salmonella* infections, only the first reported infection was considered. In analyses restricting exposure to a serovar, only the first infection of the serovar of interest was used. Considering a minimal development time of 1 year for CC formation after infection, which has been assumed previously to have a plausible relation to the infection [7, 19], people were considered at risk of CC from 1 year after reported *Salmonella* infection onwards. Hence, we defined the exposure status as a time-varying variable with three states: individuals were ‘unexposed’ (reference) until first reported infection, ‘newly exposed’ in the first year post-infection and ‘exposed’ from 1 year post-infection onwards. We excluded individuals with a diagnosed CC between January 1978 and December 1993, to reduce the risk that CC had developed before the *Salmonella* infection occurred. The outcome studied was CC (ICD-10 codes C180–C187). For the analysis, we looked at CC overall and by colon subsite: proximal colon (C180–C185) and distal colon (C186, C187). In the analyses of risk of cancer in one colon subsite individuals were not censored for cancers in the other subsite.

### Statistical analysis

Characteristics of the study population were presented descriptively. In the survival analyses, individuals were followed from birth or 1st January 1994, whichever was last. Follow-up ended at date of cancer diagnosis, death or the end of study (31st December 2016), whichever occurred first. In addition, risk time excluded periods where individuals were temporarily or permanently living outside of Denmark. Three of the potential confounders; geographical region, marital status and IBD status were time-varying variables with five (North Jutland, South Jutland, Middle Jutland, Zealand, Capital), four (unmarried, married, divorced, widowed) and two (yes, no) levels, respectively.

We used Cox proportional hazard models to estimate hazard ratios (HRs) with 95% confidence intervals (CIs) for developing CC in individuals with a history of reported *Salmonella* infection *vs.* people without such history. The main comparison of interest was exposed *vs.* unexposed; hence, all analyses show the HRs for this comparison. Besides, in the main analysis the HRs for ‘newly exposed’ *vs.* unexposed were displayed to address the potential effect of testing/diagnostic bias in symptomatic individuals with yet undiagnosed CC [20]. Attained age was used as the time scale for the baseline hazard function, which was stratified by sex, year of birth, geographical region, marital status and IBD to adjust for potential confounding effects. Additionally, we conducted analyses to examine whether HRs varied by sex, attained age (<50, 50–59, 60–69, 70–79 and ≥80 years), age at infection (<50, 50–59, 60–69, 70–79 and ≥80 years), follow-up time post-infection (2nd–5th year, 6th–10th year and >10 years) and IBD. The proportional hazards assumption of the main analysis (salmonellosis overall and CC overall) was assessed using a test for homogeneity of the HR in the age intervals; <50, 50–59, 60–69, 70–79 and ≥80 years of age. Incidence curves of CC (overall and by subsite) in the exposed *vs.* unexposed group (stratified by *Salmonella* serovar) were also generated to graphically display the comparison; incidences at all ages were calculated by weighting the number of cancers and risk days within a time span of ±5 years using a parabolic kernel. In all analyses, *P*-values <0.05 were considered statistically significant. In accordance with privacy legislation, small numbers were not displayed in tables. Statistical analysis was performed using the PHREG procedure of SAS (version 9.4).

## Results

During a total of 124.7 million person-years of follow-up, 54 902 individuals were diagnosed with CC, at a median age of 72 years (IQR: 64–80). Among those with a CC diagnosis, 278 individuals were diagnosed with CC after salmonellosis, of which 33 occurred within the first year post-infection. The median time span between infection and CC diagnosis was 7.5 years (IQR: 3.0–13.9). In the subsite-specific analyses, 29 422 individuals were diagnosed with proximal CC and 26 108 with distal CC. Table 1 shows the number of overall CC events and incidence rates (IRs) in the exposed and unexposed groups by different subgroups. The average IR of CC in the exposed group was 47.16 per 100 000 person-years at-risk, whereas in the unexposed group the IR was 44.02.

**Table 1.** IRs of CC (overall) of people with (‘exposed’) and without (‘unexposed’) a reported *Salmonella* infection per 100 000 person-years, by different subgroups

	Unexposed		Exposed^a^		Total	
	No. of events	IR^b^	No. of events	IR^b^	No. of events	IR^b^
Total Sex	54 624	44.02	278	47.16	54 902	44.04
Female	28 124	44.90	138	46.20	28 262	44.90
Male	26 500	43.14	140	48.14	26 640	43.16
Birth year						
≤1930	21 964	225.23	71	275.19	22 035	225.36
1931–1950	26 474	100.93	159	130.97	26 633	101.06
1951–1970	5728	16.27	42	25.03	5770	16.31
1971–1990	415	1.36	6	4.05	421	1.38
≥1991	43	0.19	0	0.00	43	0.19
Age group						
0–49 years	2368	2.93	17	4.48	2385	2.94
50–59 years	5942	36.06	34	40.67	5976	36.08
60–69 years	13 638	103.74	79	113.83	13 717	103.80
70–79 years	18 828	217.40	88	228.57	18 916	217.45
≥80 years	13 848	278.74	60	331.49	13 908	278.94
Marital status						
Married	30 014	61.20	164	67.57	30 178	61.23
Divorced	6011	62.28	32	65.17	6043	62.29
Not married	4117	7.10	24	8.94	4141	7.11
Widowed	14 482	196.10	58	199.31	14 540	196.11
Region						
North Jutland	6064	45.93	28	50.72	6092	45.95
South Jutland	12 390	45.83	97	65.41	12 487	45.94
Middle Jutland	11 412	40.96	48	35.93	11 460	40.93
Zealand	9132	49.90	46	48.12	9178	49.89
Capital	15 626	41.47	59	37.65	15 685	41.46
IBD status						
No	51 854	42.77	249	44.56	52 103	42.78
Yes	2770	97.97	29	94.77	2799	97.93

IR, incidence rate.

a Including both ‘newly exposed’ and exposed.

b per 100 000 person-years

### Risk of colon cancer

Adjusting for sex, year of birth, region of residence, IBD and marital status, the overall risk of CC did not differ between the exposed and unexposed groups (HR: 0.99 [95% CI 0.88–1.13]) (Table 2). Similarly, no differences were observed between these groups when stratifying by colon subsite and sex. However, within 1 year post-infection the overall risk of CC increased twofold compared to the unexposed group (HR: 2.08 [95% CI 1.48–2.93]). For the exposed group, when stratifying by serovar, an HR of 1.40 (95% CI 1.03–1.90) for cancer in the proximal colon was observed in individuals who had an infection with serovars other than *S*E and *S*T (Table 2).

**Table 2.** Risk of CC after salmonellosis, by sex, serovar and IBD status

	CC (overall)		Proximal colon		Distal colon	
	Events	HR (95% CI)^a^	Events	HR (95% CI)^a^	Events	HR (95% CI)^a^
Unexposed		ref		ref		ref
Newly exposed	33	2.08 (1.48–2.93)***	18	2.16 (1.36–3.43)**	15	1.96 (1.18–3.26)**
Exposed	245	0.99 (0.88–1.13)	144	1.09 (0.93–1.29)	102	0.87 (0.71–1.05)
*Exposed vs. unexposed*						
Sex						
Male	121	0.99 (0.83–1.18)	59	0.99 (0.77–1.28)	62	0.96 (0.75–1.24)
Female	124	1.00 (0.84–1.19)	85	1.18 (0.95–1.46)	40	0.75 (0.55–1.03)
*Salmonella* serovar						
Enteritidis	137	1.03 (0.87–1.21)	74	1.04 (0.83–1.31)	63	0.99 (0.77–1.26)
Typhimurium	43	0.74 (0.55–1.00)*	28	0.90 (0.62–1.31)	15	0.54 (0.33–0.90)*
Other serovars	65	1.17 (0.91–1.49)	42	1.40 (1.03–1.90)*	24	0.91 (0.61–1.36)
IBD						
Yes	28	1.18 (0.81–1.71)	20	1.40(0.90–2.19)	8	0.80(0.40–1.61)
No	217	0.98 (0.85–1.11)	124	1.06(0.89–1.26)	94	0.87(0.71–1.07)

HR, hazard ratio; IBD, inflammatory bowel disease; ref, reference.

a Adjusted for sex, year of birth, geographical region, IBD status and marital status.

*P-value <0.05; **P-value <0.01; ***P-value <0.001.

**Fig. 1.**
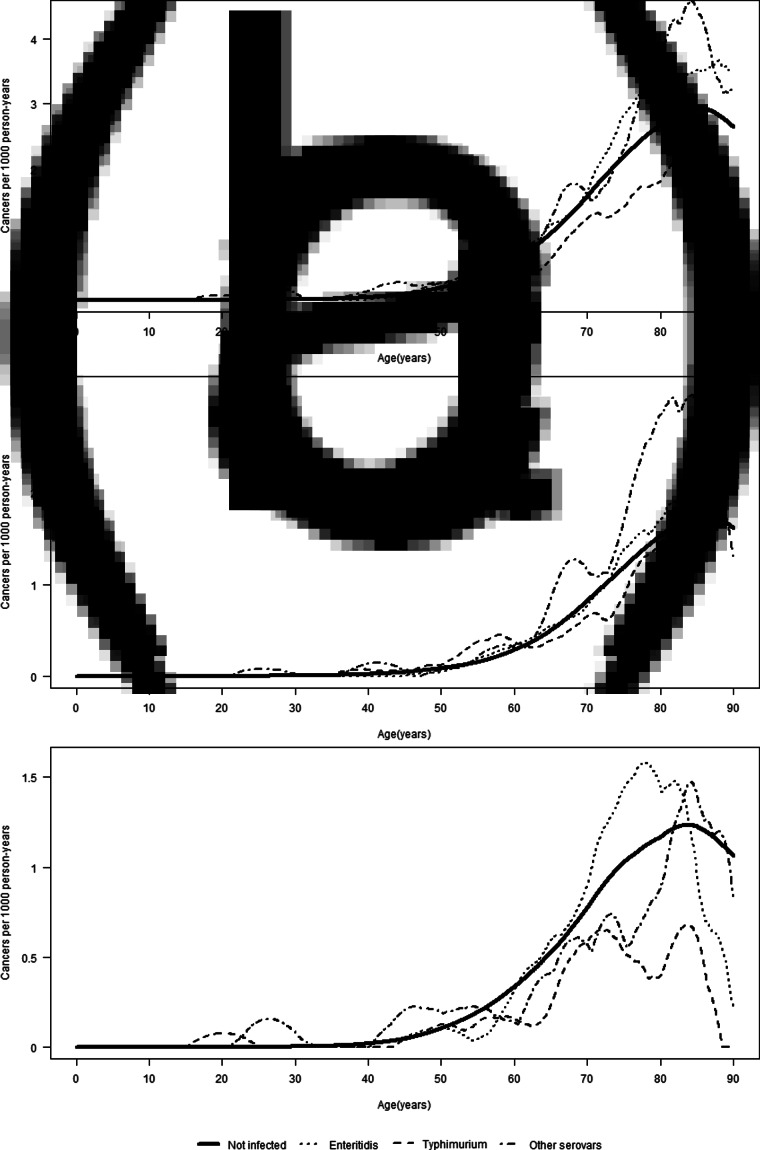
Incidence of overall (a), proximal (b) and distal (c) CC by attained age, stratified by serovar.

The association between *Salmonella* infection and CC did not vary by attained age (Table 3). A test for homogeneity of HRs in the five age groups yielded a *P*-value of 0.59. Figure 1 shows the incidence of CC (overall and per colon subsite) by attained age for the different serovars. For both proximal and distal CC, the IRs were the lowest for *S*T in people aged above 60 years as compared to *S*E and other serovars. In the age-stratified analyses, a 1.87-fold (95% CI 1.00–3.50) increased risk of distal CC was observed in the exposed group aged 0–49 years (for *Salmonella* overall). For the proximal colon, the highest HR, although not significant, was also observed in the age group 0–49 years among those infected with other serovars (HR: 1.75; 95% CI 0.56–5.44). The estimated association between *Salmonella* and CC risk did not vary much by age at infection (Table 4). The median observed ages at infection of different serovars in the total cohort were 37 years (IQR: 16–54) for *S*E, 30 years (IQR: 9–52) for *S*T and 32 years (IQR: 16–54) for other serovars.

**Table 3.** Risk of CC ≥1 year after salmonellosis, by attained age group and serotype

	CC (overall)	Proximal colon	Distal colon
	HR (95% CI)^a^	HR (95% CI)^a^	HR (95% CI)^a^
*Salmonella* (total)			
Unexposed	ref	ref	ref
<50 years	1.39 (0.85–2.27)	0.96 (0.43–2.14)	1.87 (1.00–3.50)*
50–59 years	0.90 (0.62–1.32)	1.32 (0.83–2.11)	0.54 (0.28–1.05)
60–69 years	0.97 (0.77–1.23)	1.08 (0.78–1.48)	0.89 (0.63–1.25)
70–79 years	0.93 (0.75–1.17)	0.97 (0.72–1.30)	0.87 (0.62–1.22)
≥80 years	1.09 (0.84–1.43)	1.24 (0.89–1.71)	0.83 (0.52–1.34)
*Salmonella* Enteritidis			
Unexposed	ref	ref	ref
<50 years	0.70 (0.26–1.88)	0.34 (0.05–2.39)	1.09 (0.35–3.40)
50–59 years	0.62 (0.33–1.15)	0.95 (0.45–2.01)	0.34 (0.11–1.04)
60–69 years	1.06 (0.78–1.44)	0.99 (0.63–1.55)	1.11 (0.73–1.69)
70–79 years	1.10 (0.84–1.46)	1.00 (0.67–1.47)	1.19 (0.80–1.77)
≥80 years	1.18 (0.83–1.67)	1.34 (0.88–2.03)	0.87 (0.47–1.63)
*Salmonella* Typhimurium			
Unexposed	ref	ref	ref
<50 years	1.39 (0.52–3.71)	1.23 (0.31–4.96)	1.57 (0.39–6.30)
50–59 years	1.24 (0.65–2.40)	1.83 (0.82–4.09)	0.75 (0.24–2.33)
60–69 years	0.68 (0.38–1.24)	0.88 (0.42–1.85)	0.49 (0.18–1.30)
70–79 years	0.60 (0.34–1.05)	0.73 (0.36–1.45)	0.43 (0.16–1.14)
≥80 years	0.59 (0.28–1.25)	0.70 (0.29–1.69)	0.42 (0.10–1.66)
Other *Salmonella* serovars			
Unexposed	ref	ref	ref
<50 years	2.61 (1.30–5.25)**	1.75 (0.56–5.44)	3.65 (1.51–8.86)**
50–59 years	1.19 (0.60–2.39)	1.62 (0.67–3.90)	0.82 (0.26–2.55)
60–69 years	1.03 (0.64–1.67)	1.45 (0.82–2.55)	0.73 (0.33–1.62)
70–79 years	0.89 (0.55–1.43)	1.14 (0.65–2.01)	0.57 (0.24–1.36)
≥80 years	1.44 (0.87–2.40)	1.57 (0.84–2.93)	1.18 (0.49–2.85)

HR, hazard ratio; ref, reference.

a Adjusted for sex, year of birth, geographical region, IBD status and marital status.

*P-value <0.05; **P-value <0.01; ***P-value <0.001.

**Table 4.** Risk of CC ≥1 year after salmonellosis, by age group at infection and serotype

	CC (overall)	Proximal colon	Distal colon
	HR (95% CI)^a^	HR (95% CI)^a^	HR (95% CI)^a^
*Salmonella* (total)			
Unexposed	ref	ref	ref
<50 years	1.10 (0.84–1.45)	1.07 (0.72–1.60)	1.12 (0.76–1.63)
50–59 years	0.91 (0.71–1.16)	1.02 (0.74–1.42)	0.77 (0.53–1.13)
60–69 years	0.95 (0.75–1.20)	1.08 (0.80–1.45)	0.80 (0.55–1.17)
70–79 years	1.11 (0.84–1.47)	1.22 (0.86–1.73)	0.92 (0.58–1.47)
≥80 years	0.91 (0.50–1.65)	1.13 (0.56–2.26)	0.57 (0.18–1.77)
*Salmonella* Enteritidis			
Unexposed	ref	ref	ref
<50 years	0.90 (0.60–1.36)	0.91 (0.50–1.64)	0.89 (0.51–1.57)
50–59 years	0.90 (0.65–1.26)	0.87 (0.54–1.39)	0.93 (0.58–1.47)
60–69 years	1.08 (0.80–1.46)	1.08 (0.72–1.61)	1.06 (0.68–1.67)
70–79 years	1.27 (0.89–1.82)	1.31 (0.83–2.09)	1.15 (0.65–2.02)
≥80 years	1.03 (0.46–2.30)	1.19 (0.44–3.19)	0.76 (0.19–3.09)
*Salmonella* Typhimurium			
Unexposed	ref	ref	ref
<50 years	1.16 (0.67–2.00)	1.47 (0.74–2.95)	0.86 (0.36–2.07)
50–59 years	0.88 (0.51–1.52)	1.08 (0.54–2.16)	0.67 (0.28–1.62)
60–69 years	0.51 (0.27–0.99)	0.63 (0.28–1.41)	0.36 (0.12–1.13)
70–79 years	0.52 (0.23–1.16)	0.59 (0.22–1.57)	0.41 (0.10–1.65)
≥80 years	0.64 (0.16–2.58)	1.06 (0.27–4.28)	–
Other *Salmonella* serovars			
Unexposed	ref	ref	ref
<50 years	1.52 (0.91–2.52)	1.00 (0.42–2.41)	2.00 (1.08–3.73)*
50–59 years	0.93 (0.56–1.54)	1.33 (0.73–2.40)	0.50 (0.19–1.32)
60–69 years	1.08 (0.68–1.71)	1.54 (0.91–2.61)	0.64 (0.27–1.54)
70–79 years	1.43 (0.85–2.42)	1.73 (0.93–3.23)	0.95 (0.36–2.55)
≥80 years	0.94 (0.30–2.93)	1.06 (0.26–4.24)	0.73 (0.10–5.22)

HR, hazard ratio; ref, reference.

a Adjusted for sex, year of birth, geographical region, IBD status and marital status.

*P-value <0.05; **P-value <0.01; ***P-value <0.001.

There was no significant effect of follow-up time post-infection on CC risk; the HRs of proximal CC for people infected with other serovars were 1.62 (95% CI 0.96–2.74), 1.47 (95% CI 0.85–2.53) and 1.20 (95% CI 0.72–1.90) at 1–5 years, 5–10 years and >10 years post-infection, respectively (Table 5). With regards to the potential effect modification of IBD on CC risk, the HR for overall CC was not significantly higher for people with underlying IBD (HR 1.18; 95% CI 0.81–1.71) (Table 2).

**Table 5.** Risk of CC ≥1 year after salmonellosis, by serovar and time post-infection

	CC (overall)	Proximal colon	Distal colon
	HR (95% CI)^a^	HR (95% CI)^a^	HR (95% CI)^a^
*Salmonella* (total)			
Unexposed	ref	ref	ref
1–5 years	1.09 (0.85–1.38)	1.29 (0.95–1.75)	0.88 (0.60–1.29)
5–10 years	0.96 (0.76–1.22)	1.03 (0.75–1.42)	0.86 (0.60–1.24)
>10 years	0.97 (0.80–1.16)	1.03 (0.81–1.32)	0.87 (0.65–1.16)
*Salmonella* Enteritidis			
Unexposed	ref	ref	ref
1–5 years	1.22 (0.88–1.70)	1.30 (0.83–2.03)	1.12 (0.69–1.83)
5–10 years	0.98 (0.71–1.36)	1.02 (0.66–1.58)	0.91 (0.56–1.48)
>10 years	0.97 (0.76–1.23)	0.95 (0.68–1.33)	0.97 (0.68–1.38)
*Salmonella* Typhimurium			
Unexposed	ref	ref	ref
1–5 years	0.86 (0.51–1.45)	0.92 (0.46–1.83)	0.78 (0.35–1.73)
5–10 years	0.45 (0.23–0.90)*	0.64 (0.29–1.42)	0.24 (0.06–0.95)*
>10 years	0.87 (0.57–1.34)	1.09 (0.64–1.83)	0.61 (0.29–1.29)
Other *Salmonella* serovars			
Unexposed	ref	ref	ref
1–5 years	1.05 (0.65–1.69)	1.62 (0.96–2.74)	0.52 (0.20–1.39)
5–10 years	1.45 (0.97–2.17)	1.47 (0.85–2.53)	1.41 (0.78–2.54)
>10 years	1.04 (0.70–1.55)	1.20 (0.72–1.99)	0.83 (0.43–1.60)

HR, hazard ratio; ref, reference.

a Adjusted for sex, year of birth, geographical region, IBD status and marital status.

*P-value <0.05; **P-value <0.01; ***P-value <0.001.

## Discussion

We assessed the risk of CC after reported *Salmonella* infection in a 23-year follow-up of the entire Danish population. The risk of CC in individuals with reported salmonellosis was compared to the risk in individuals without a reported salmonellosis, accounting for potential confounding and modifying effects of age, sex, IBD and follow-up time post-infection. Overall, we observed no increased risk of CC among salmonellosis cases. The only significantly increased risk of CC concerned the proximal colon after infection with serovars other than *S*E and *S*T. The proximal part of the colon is the subsite of primary interest, as exposure to *Salmonella* is highest in this part of the large intestine located directly after the ileum, where *Salmonella* typically establishes infection. CC risk was highest at an attained age of <50 years for those infected with other serovars. For *S*E, the estimated HRs increased with increasing age, whereas for *S*T they decreased with increasing age, which may be due to differences in age-specific reporting between these serovars.

In a recent Dutch study, a statistically significantly increased risk of cancer in the proximal colon was found among individuals with a history of *S*E infection [7]. This result was not confirmed in the current study, indicating that the previously observed association between *S*E infection and proximal CC is not generalisable to other study populations. On the one hand, the inconsistent findings might be explained by a more complex causal mechanism than originally anticipated and the existence of situational differences, but may also represent a chance finding. Indeed, our results seem to indicate a possible scenario of increased risk of proximal CC associated with infection with a *Salmonella* serovar other than *S*E or *S*T, but it could also be the result of type I error due to multiple hypothesis testing.

For surveillance design reasons, the two studies used different types of analyses and effect measures. The Dutch *Salmonella* surveillance system covers approximately 64% of the population; therefore, the risk of CC in individuals with a reported salmonellosis was compared to the baseline CC risk in the general Dutch population, expressed as standardised incidence ratios [7]. The Danish surveillance system covers the whole population, which allowed us to compare the risk of CC in people with those without a reported salmonellosis using Cox regression. Yet, both studies used individual-level data and the inclusion criteria were comparable. Apart from the aforementioned possibility of a chance finding, other and largely unknown factors might underlie this dissimilarity including, for instance, different serovar distributions and populations exposed to them. Disease outcomes (e.g. severity, antimicrobial resistance, etc.) and epidemiology (e.g. sources, modes of transmission, high-risk groups, etc.) differ by serovar, partly due to differences in exposure but also potential factors related to virulence, invasiveness and toxins of the bacterium itself [21]. The estimated number of *Salmonella* infections in Denmark is somewhat higher compared to the Netherlands, with respectively 18.1 and 15.8 infections per 10 000 inhabitants in Denmark and the Netherlands in 2017 [12, 22]. In both the Netherlands and Denmark, *S*E accounts for a substantial part (25–30%) of the salmonellosis cases [11, 23]. The successful implementation of a *Salmonella* control programme in the poultry production chain led to a marked reduction of domestically acquired human *S*E infections in Denmark since 1998, with most infections nowadays being attributable to foreign travel (78.2% in 2016) [11, 24]. In contrast, most *S*E infections in the Netherlands remain domestically acquired; therefore, the groups of people infected with *S*E might not be fully comparable in terms of, e.g. general health status, lifestyle, socio-economic status, ethnicity and possible co-morbidities. Besides, with regards to serovars other than *S*E and *S*T, different distribution and exposure patterns, as well as specific strains, might also have contributed to these differences, as the genetic makeup of the strains themselves might be associated with their ability to transform [21].

It has been shown experimentally that *Salmonella* infection of pre-transformed fibroblasts and organoids induces full cell transformation [4]. Development from a pre-malignant state to an advanced carcinoma takes several years; however, *Salmonella* infection is likely to accelerate this process [4, 25 ]. The results of a sub-analysis showed a twofold increased risk of CC (overall and per subsite) for individuals within the first year post-infection. Even though *Salmonella* could accelerate transformation, tumour development in less than 1 year seems implausible. We therefore consider this observation to reflect testing/diagnostic bias rather than the transformation capacity of *Salmonella*. Undiagnosed CC patients often present at their general practitioner (GP) with nonspecific symptoms resembling gastroenteritis, such as diarrhoea and frequent bowel movements. In a Danish cohort, it was shown that CC patients had significantly more GP consultations in the 9 months prior to the cancer diagnosis [17]. A similar pattern was observed in another Danish cohort study that examined the risk of IBD after a *Salmonella-*positive stool test. An increased risk of IBD was observed in the first year after *Salmonella* infection; however, this was even more pronounced in the first year following a negative stool test [9]. The association we found in the first year post-infection is compatible with these prior observations. An alternative hypothesis might be that people in an early stage of cancer are more susceptible to *Salmonella* infection due to dysbiosis or other changes in the gut microbiome [26], so the association observed in the first year after infection might also reflect reverse causality. Still, both the testing/diagnostic bias and the increased susceptibility could co-exist in people with an early-stage pre-diagnosed CC.

This study has some limitations. First, mainly severe infections, outbreak-related infections or infections with a suspected foreign source are included, as most people do not present at their GP with mild and self-limiting gastrointestinal complaints. Hence, we could not assess whether multiple mild *Salmonella* infections that are undiagnosed and unreported contribute to CC risk or not. This could be the subject of another study using, for instance, serology to measure the magnitude of exposure to *Salmonella* regardless of reporting bias. Second, in the Dutch cohort, the risk of cancer was only significantly increased for enteric infections and not for invasive (bloodstream) infections [7], but we were not able to address this observation in the current study. Third, we were not able to control for some of the main risk factors for CC, such as obesity, smoking and alcohol consumption. Although considering these variables would be relevant to explain CC risk along with the studied *Salmonella* infection, this would require a different study design as these types of time-varying variables are not generally present in national health registries.

In conclusion, the current study found no unusual CC risk associated with previously reported *Salmonella* infection overall. Therefore, although there is growing experimental evidence for a potential role of *Salmonella* in CC development, notified *Salmonella* infections do not appear to be an important driver of CC risk in the studied population. Indeed, the previously observed epidemiological association between *S*E infection and proximal CC was not confirmed here. The explanation for these differences, if not merely occurring by chance, is unclear.

## Data Availability

All data relevant to the study are included in the article or uploaded as supplementary information.
